# Stability and suitability of genotypes and environment to Ascochyta blight of chickpea

**DOI:** 10.3389/fpls.2023.1006099

**Published:** 2023-03-28

**Authors:** Mamta Sharma, U. S. Sharath Chandran, Upasana Rani, Sudhir K. Singh, Ashwani K. Basandrai, Daisy Basandrai

**Affiliations:** ^1^ Precision Phenotyping for Biotic-Abiotic Stresses and Nutrition, Accelerated Crop Improvement, International Crops Research Institute for the Semi-Arid Tropics, Patancheru, India; ^2^ Center of Excellence on Climate Change Research for Plant Protection, International Crops Research Institute for the Semi-Arid Tropics, Patancheru, India; ^3^ Department of Plant Breeding and Genetics, Punjab Agricultural University, Ludhiana, India; ^4^ Organic Farming Research Centre, Chatha, Sher-e-Kashmir University of Agricultural Sciences and Technology, Jammu, India; ^5^ Department of Plant Pathology, CSK Himachal Pradesh Agricultural University, Palampur, India; ^6^ Department of Genetics and Plant Breeding, CSK Himachal Pradesh Agricultural University, Palampur, India

**Keywords:** Ascochyta blight, chickpea, multi-environment testing, G x E interaction, GGE biplots, principal component analysis

## Abstract

Ascochyta blight (AB) is a major biotic constraint to chickpea production internationally. The disease caused by the phytopathogenic fungus *Ascochyta rabiei* is highly favored by prolonged spells of low temperature and high humidity. The disease scenario is expected to aggravate in the near future as a result of rapidly changing climatic conditions and the emergence of fungicide-resistant pathogen strains. Tapping into host–plant resistance is the most logical way to preempt such a crisis. Presently, high levels of stable resistance against AB are yet to be identified from the chickpea gene pool. The present study was aimed at facilitating this process through multi-environment testing of chickpea genotypes. Using the GGE biplot analysis method, we could identify three genotypes, viz., ICCV 16508, ICCV 16513, and ICCV 16516, from the International Ascochyta Blight Nursery, which showed consistent moderate resistance reactions across all the tested environments. Moreover, we were able to evaluate the test locations for their suitability to support AB screening trials. Ludhiana and Palampur locations were identified as the most ideal for continual screening in the future. Controlled environment screening at the ICRISAT location offered to reduce large plant populations to small meaningful sizes through initial screening under controlled environment conditions. This study will further improve the scope of phenotyping and sources of stable resistance to be utilized in future AB resistance breeding programs.

## 1 Introduction

Chickpea (*Cicer arietinum* L.) is the third most important cool season food legume cultivated in several countries. The global annual production of chickpeas was 14.25 million metric tonnes in 2019, of which India alone contributes to nearly 65% of the total production (FAOSTAT, 2019). In the major chickpea-growing belts of India, Ascochyta blight (AB) caused by the fungus *Ascochyta rabiei* (Pass.) Labr. has turned out to be a major biotic constraint. It is a devastating disease prevalent in regions predisposed to prolonged low temperature (15°C–25°C) and high humidity (>150-mm rainfall) conditions (Pande et al., 2011). In India, the northern states (Himachal Pradesh, Punjab, Haryana, Jammu, and Kashmir) are reported to have higher AB prevalence owing to the conducive weather conditions during its chickpea-growing seasons (Baite and Dubey, 2018; Manjunatha et al., 2018). Mono-cropping and intensification of chickpea production have led to large-scale epidemics (Gan et al., 2006) causing severe reductions in seed quality and yield. In some cases, where conditions are highly favorable, AB accounts for up to 100% of crop losses (Pande et al., 2005; Tivoli et al., 2006).

The sources of stable AB resistance donors in chickpeas are still scarce (Sharma and Ghosh, 2016), partly due to the rapid adaptation of *A. rabiei* against the plant defense mechanisms (Gayacharan et al., 2020) and partly due to the high expense incurred in large-scale multi-location testing of the genetically diverse chickpea germplasm and breeding lines (Pande et al., 2013). The success of breeding programs is highly dependent on the availability of donor genotypes having consistent and stable performance over multi-year and multi-location environments (Parihar et al., 2017). This is because quantitative traits like disease resistance (or susceptibility) in a plant are highly influenced by the current environment relative to spatio-temporal climatic variations. Thus, evaluating the individual and combined impacts of the genotypes and environment on AB disease resistance will play a key role in identifying superior resistance in chickpeas. To achieve this, multivariate techniques (Pande et al., 2013; Poole et al., 2013) including correlation models that focus on the relationship between the disease severity (DS) and environmental factors (Smiley and Yan, 2009) can be widely used. A more recent method, the graphical GGE (genotype main effect (G) plus genotype by environment interaction (GE)) biplot technique, is now being used widely to evaluate the stability of genotypes, environment, and consequent genotype by environment (G × E) interaction from multi-environment trials (Parihar et al., 2017).

Integrated approaches including the use of resistant cultivars and foliar fungicides are the best approach to managing AB (Owati et al., 2017). Currently, we are more dependent on the use of fungicides than on host plant resistance for the control of this disease. However, several reports have shown the emergence of fungicide-resistant *A. rabiei* strains that have the potential to make chemicals less effective and allow extreme epidemic incidences in major chickpea-growing areas (Chang et al., 2007; Wise et al., 2009; Owati et al., 2017). The best course of action would be to strengthen the plant-breeding activities in this direction for developing AB-resistant chickpea lines. Multi-year, multi-location trials followed by suitable stability analysis are highly useful methods in disease resistance breeding. Therefore, the present study was aimed at 1) identifying durable resistance in chickpeas against AB and 2) identifying suitable locations that support the natural screening of chickpeas against AB.

## 2 Materials and methods

### 2.1 Plant material and controlled environment screening

A total of 160 chickpea genotypes (ICRISAT germplasm accessions) were evaluated for AB resistance under controlled environment conditions in the year 2016/2017 at the ICRISAT location, following the standard seedling screening technique outlined by Pande et al., 2011; Pande et al., 2013). The experiment was conducted in a randomized complete block design (RCBD) with three replications. Briefly, the seedlings were raised in sterilized river sand and vermiculite mixture (10:1 ratio) in plastic trays under greenhouse conditions of 25°C ± 2°C for 10 days. A single tray accommodated 10 genotypes (nine test genotypes and one susceptible check) with eight seeds per genotype per test row. Ten-day-old seedlings were shifted to the growth chambers maintained at 20°C ± 1°C with a 12-h photoperiod and acclimatized for 24 h. The highly virulent *A. rabiei* isolates (Accession No. ITCC 6651 (Pande et al., 2011)) were mass multiplied on sterilized kabuli chickpea, conidia were collected in sterilized water, and concentration was adjusted using a hemocytometer. The artificial inoculation of the seedlings was performed by spraying the foliage with the spore suspension (5 × 10^4^ conidia/ml) until run-off. Initially, a continuous relative humidity (RH) of 100% was maintained for 96 h, after which it was reduced to 6–8 h per day for the next 7 days. On day 8 after inoculation, DS was assessed on the seedlings based on a 1–9 rating scale (where ratings 1 and 9 represent “asymptomatic” and “highly susceptible” classes, respectively). Genotype ICC 4991 served as the susceptible control check line.

The controlled environment screening was repeated to remove all highly susceptible (DS rating 9) genotypes before establishing an International Ascochyta Blight Nursery (IABN) with the selected candidate genotypes (**Table 1**).

**Table 1:** Mean AB severity (1–9 scale) of IABN across four locations during 2017/2018 and 2018/2019.

Sl no.	Genotype	Code	Environment								Mean
			IC17	IC18	JA17	JA18	LU17	LU18	PA17	PA18	
1	ICCV 16501	G1	6.0	7.0	5.0	4.0	9.0	9.0	6.0	8.0	6.8
2	ICCV 16502	G2	6.0	5.5	4.0	8.0	8.0	9.0	6.0	5.0	6.4
3	ICCV 16503	G3	5.5	5.0	5.0	3.0	8.5	9.0	6.0	8.5	6.3
4	ICCV 16505	G4	5.0	5.5	7.0	7.0	3.5	5.5	4.0	3.0	5.1
5	ICCV 16506	G5	5.0	6.0	7.0	6.0	8.0	9.0	5.5	8.0	6.8
6	ICCV 16507	G6	5.0	7.0	5.0	7.0	6.0	9.0	4.5	6.0	6.2
7	ICCV 16508	G7	4.0	5.0	6.0	7.0	3.5	5.0	4.5	2.5	4.7
8	ICCV 16509	G8	4.5	8.0	7.0	7.0	7.0	7.5	6.0	5.5	6.6
9	ICCV 16510	G9	4.0	8.0	6.0	8.0	4.5	5.0	5.0	4.5	5.6
10	ICCV 13616	G10	5.0	7.0	5.0	7.0	6.5	8.5	5.5	3.5	6.0
11	ICCV 13622	G11	5.5	7.0	5.0	6.0	2.0	3.0	3.5	3.0	4.4
12	ICCV 16511	G12	6.5	7.0	5.0	7.0	2.5	3.0	3.5	3.0	4.7
13	ICCV 16512	G13	7.0	8.0	6.0	7.0	3.5	6.0	4.0	3.5	5.6
14	ICCV 16513	G14	6.5	6.0	6.0	3.0	3.0	4.5	3.5	2.5	4.4
15	ICCV 16516	G15	5.5	7.0	6.0	5.0	5.5	6.5	4.5	2.0	5.3
16	ICCV 16517	G16	5.5	7.0	5.0	7.0	5.5	3.5	4.0	2.0	4.9
17	ICCV 16518	G17	6.0	6.5	7.0	7.0	9.0	9.0	6.0	7.0	7.2
18	ICCV 16519	G18	7.0	7.0	3.0	7.0	9.0	9.0	7.0	7.5	7.1
19	ICCV 16520	G19	7.0	7.5	6.0	6.0	8.5	9.0	6.0	8.5	7.3
20	ICC 4991*	G20	9.0	9.0	7.7	8.5	8.0	8.5	6.0	8.0	8.1
Mean			5.8	6.8	5.7	6.4	6.1	6.9	5.1	5.1	

AB, Ascochyta blight; IABN, International Ascochyta Blight Nursery.

^*^ Highly susceptible check genotype.

### 2.2 Multi-environment testing

The IABN was further evaluated in field conditions at three locations, viz., Ludhiana (Punjab), Palampur (Himachal Pradesh), and Jammu, and in the controlled environment facility of ICRISAT (Hyderabad, Telangana), for two crop seasons during 2017/2018 and 2018/2019. Chickpea-growing regions of Punjab and Himachal Pradesh were selected, as they have been frequently reported to show high AB incidences in the past years under favorable environmental conditions (Basandrai et al., 2007; Pande et al., 2013), while Jammu was considered for the trial experiments, taking into account the sporadic occurrences in past years (Baite et al., 2016) and the presence of favorable agro-climatic conditions. ICRISAT was selected for the controlled environment growth chambers that facilitated optimal conditions for AB disease expression.

Both the controlled environment testing and the field trials were conducted following the methodology outlined by Pande et al. (2013). The screening protocol for the IABN at ICRISAT under a controlled environment was the same as described above. The field trials were laid out in RCBDs with two replications at each location. Plants of the test genotypes were grown in 4-m-long test lines with 30- and 10-cm inter-row and inter-plant spacing, respectively. At the onset of flowering, a spore suspension (1 × 10^5^ conidia/ml) of the virulent *A. rabiei* isolate (as described above) was sprayed at the rate of 5 L/100 m^2^. The spraying was repeated twice in a 10-day interval to ensure sufficient inoculum potential for disease development. High levels of humidity (>85%) were maintained by sprinklers irrigating the nursery for 10 min every hour during the daytime. The DS was assessed based on the 1–9 rating scale described above.

### 2.3 Statistical analysis

Analysis of variance (ANOVA) was conducted to explain the partition of variation as a result of genotypes, environment, and G × E interaction. Here, the test locations together with the year of the experiment constituted the factor environment. The stability of both genotypes and locations was determined using the GGE biplot analysis by Yan et al. (2001). The biplots were constructed by plotting the first principal component (PC1) against the second principal component (PC2) resulting in the singular value decomposition (SVD) of the environment-centric data and estimating each element of the matrix with the following formula:


$$Y_{ij}=\mu +e_{j}+\sum_{n=1}^{N}\lambda_{n}\gamma_{in}\delta_{jn}+\epsilon_{ij},$$

where *Y_ij_* is the mean incidence of the *i*th genotype in the *j*th environment; *µ* is the grand mean for all environments; *e_j_* is the environment deviations from the grand mean; *λ_n_* is the eigenvalue of the principal component analysis axis; *γ_in_* and *δ_jn_* are the genotype and environment principal components score for axis *n*; *N* is the number of principal components retained in the model; *ϵ_ij_* is the residual effect ~*N*(0, *σ* ^2^).

The data on the genotypic response toward AB infection across the tested locations were analyzed without scaling (“Scaling = 0”) so as to generate tester-centered (“Centering = 2”) GGE biplots (Singh et al., 2020). The ANOVA and GGE biplots were generated using the “METAN” package in the RStudio software.

## 3 Results

### 3.1 Controlled environment screening

The preliminary controlled environment screening of 160 chickpea genotypes at ICRISAT in 2016 revealed a broad genotypic response against the AB disease reaction. Among these, 19 genotypes that presented a moderate-to-susceptible reaction (DS rating between 4 and 7) were selected for multi-environment screening, while the highly susceptible genotypes (DS rating > 7) were removed from further studies. High levels of resistance (DS rating< 4) were not found during the preliminary screening. Ultimately, an IABN (**Table 1**) was established with 20 genotypes (G1–G20) that included 19 moderately resistant to susceptible genotypes and a highly susceptible check line, ICC 4991.

### 3.2 Multi-environment testing

The effects of genotype, environment, and genotype × environment were found to be significant (p< 0.05) for DS as revealed by the analysis of variance (**Table 2**). The response of the 20 chickpea genotypes toward AB infection and the overall disease spectrum was highly variable across the four test locations over 2 years. Irrespective of years, the mean DS of the susceptible check G29 (ICC 4991) was found to be 8.1 averaged across the three locations (min. 6.0 and max. 9.0), while that of the 20 genotypes was 6.3 at ICRISAT, 6.0 at Jammu, 6.5 at Ludhiana, and 5.1 at Palampur (**Table 1**).

**Table 2:** Analysis of variance for AB severity of IABN across four locations during 2017/2018 and 2018/2019.

Source of variation	Degree of freedom	Sum of squares	Mean sum of squares	F value	Pr (>F)
Genotype	19	352.1978	18.53673	26.88052	3.83E−39
Environment	7	141.5745	20.22492	29.32861	2.53E−25
G × E	133	566.3149	4.258007	6.17463	1.63E−25
Residuals	152	104.8188	0.689597	–	–
CV (%)	13.91789				

AB, Ascochyta blight; IABN, International Ascochyta Blight Nursery.

**Figure 1:**
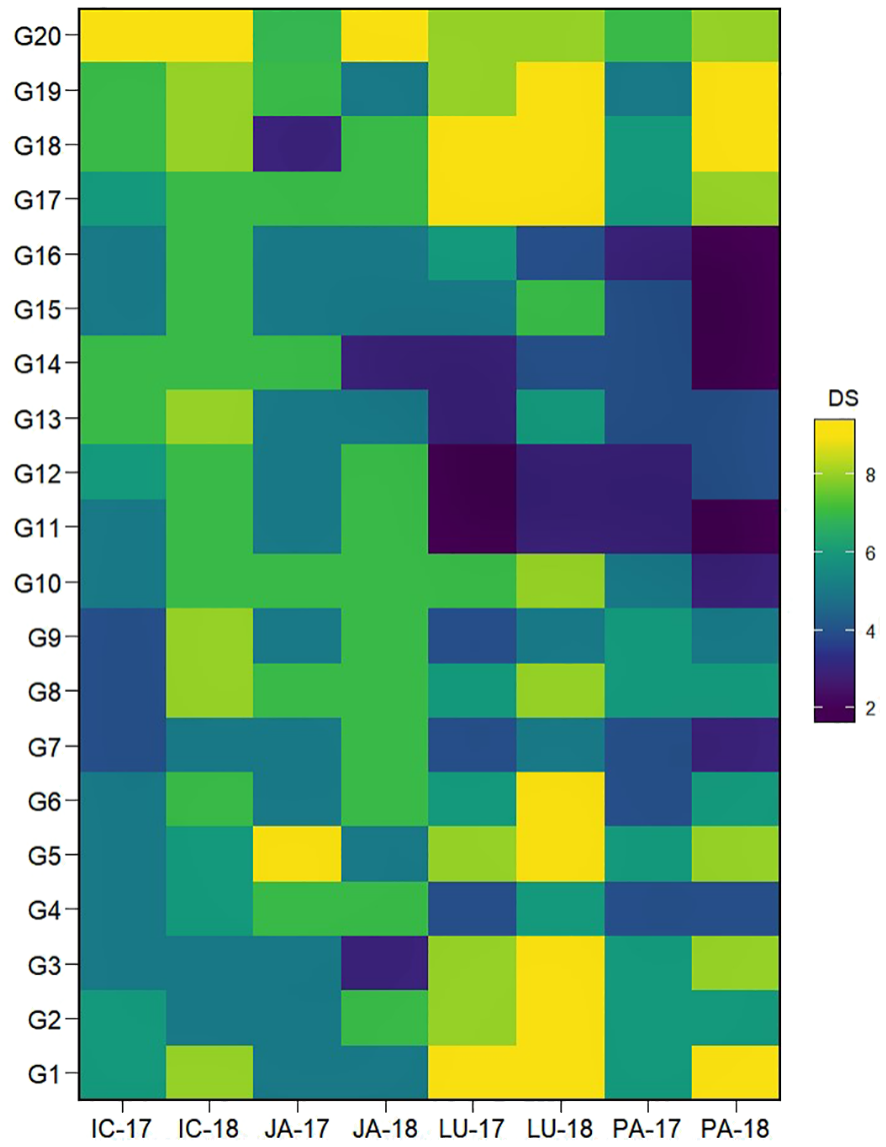
Heatmap visualization of the AB severity in IABN across four locations during 2 years. The x-axes show the tested environments. Locations are denoted as IC- (ICRISAT), JA- (Jammu), LU- (Ludhiana), and PA- (Palampur). Years are denoted as -17 (year 2017/2018) and -18 (year 2018/2019). The y-axes show the tested genotypes. The plot legend DS or disease severity depicts the 1–9 scale for AB severity rating in color. AB, Ascochyta blight; IABN, International Ascochyta Blight Nursery.

The individual performance of many genotypes varied greatly over the different locations as well as between the years tested (**Figure 1**). Based on the mean DS across the different locations over both years, seven genotypes, viz., G4, G7, G11, G12, G14, G15, and G16, that exerted a mean disease rating of ≤5.5 were identified as moderately resistant. Among them, G11 and G14 displayed a lower DS average of 4.4. The influence of the varying environments was clearly evident in the genotypic reaction to AB severity; for example, genotypes G11 and G14, which displayed resistance at Ludhiana and Palampur, showed susceptible reactions at ICRISAT and Jammu.

### 3.3 Stability of factors G and E

**Figure 2:**
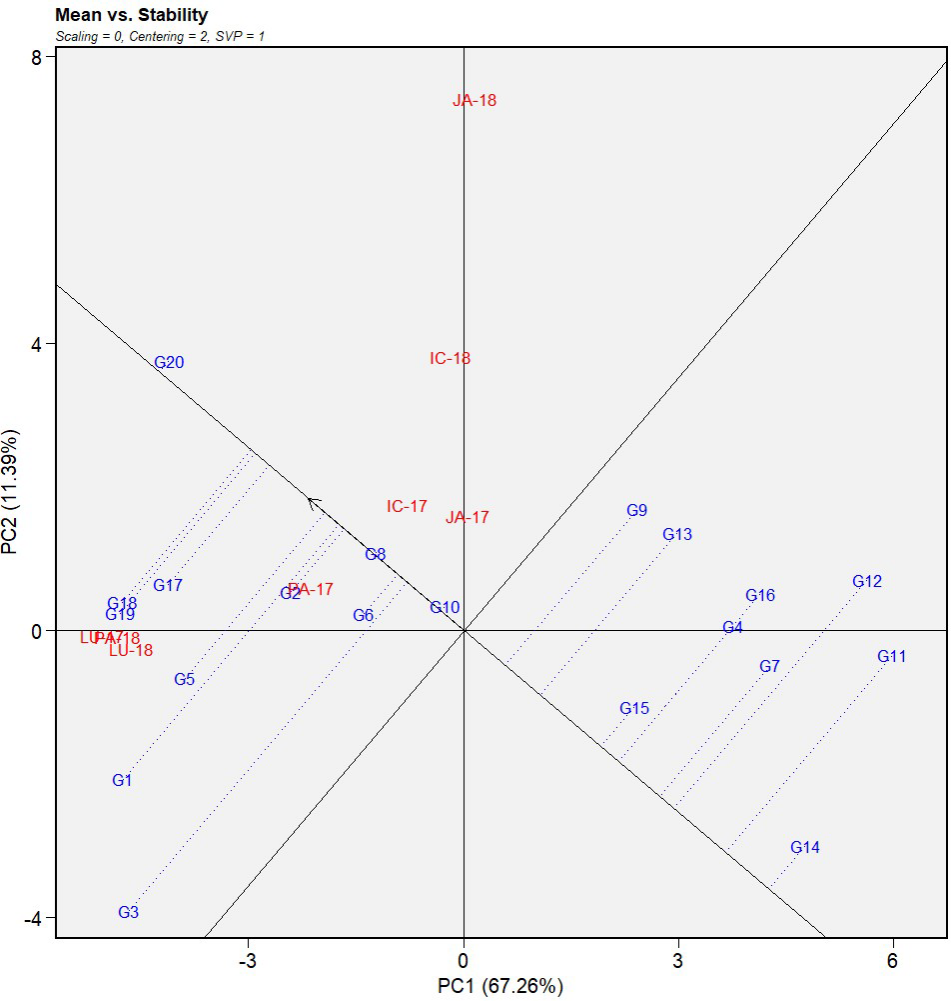
Mean *vs.* stability view of GGE biplot of IABN against AB severity across four locations during 2 years. The data were centered through environments (centering = 2) without scaling (scaling = 0). Locations are denoted as IC- (ICRISAT), JA- (Jammu), LU- (Ludhiana), and PA- (Palampur). Years are denoted as -17 (year 2017/2018) and -18 (year 2018/2019). IABN, International Ascochyta Blight Nursery; AB, Ascochyta blight.

**Figure 3:**
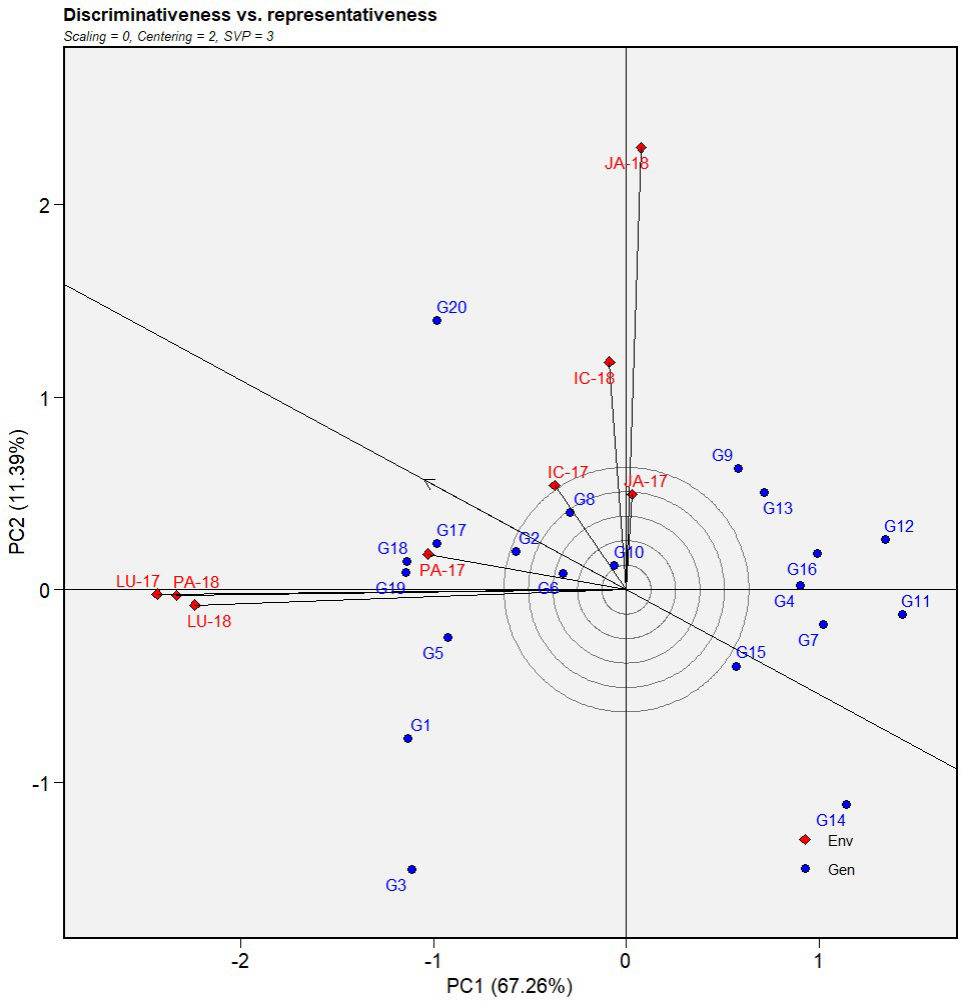
Discriminativeness *vs.* representativeness view of GGE biplot of IABN against AB severity across four locations during 2 years. The data were centered through environments (centering = 2) without scaling (scaling = 0). Locations are denoted as IC- (ICRISAT), JA- (Jammu), LU- (Ludhiana), and PA- (Palampur). Years are denoted as -17 (year 2017/2018) and -18 (year 2018/2019). IABN,International Ascochyta Blight Nursery; AB, Ascochyta blight.

The first two principal components explained 76.46% of the total variation of the environment-focused IABN data from the multi-environment testing. Here, PC1 (DS) and PC2 (resistance stability) accounted for 67.26 and 11.39% of the total variation, respectively (**Figures 2**, **3**).

#### 3.3.1 Evaluation of factor G

The mean performance of the 20 genotypes and their stability across the tested locations were assessed using the average environment coordination (AEC) view of the genotype-focused mean *vs.* stability biplot (**Figure 2**). The average environment was demarcated by the single arrowhead line, otherwise known as the AEC abscissa, passing through the biplot origins. The arrow in this axis pointed in the direction of increasing mean performance. The genotypes that occurred in the direction of the AEC abscissa from the origins indicated higher DS and consequently poor performance of the genotypes, whereas those in the opposite direction signified a more resistant reaction. Genotypes G20 and G14 displayed the highest and lowest DS, respectively, as evident from their positions in the two extremes of the average environment AEC abscissa. Overall, nine genotypes with lower DS means were found to occur in the opposite direction of the AEC abscissa from the biplot origin.

The overall stability of the genotypes in the multi-environments was also estimated by projecting the genotypes onto the AEC abscissa (**Figure 2**). The further the genotypes projected from the AEC abscissa, the lower the stability of its reaction throughout the environments and vice versa. Genotype G3 displayed the longest projection and is thereby the least stable or the most inconsistent performer among the 20 genotypes tested. G8, G10, and G20 with mean susceptible reactions gave the least projection onto the axis implying very high stability across the tested environments. In our study, G14 and G15 were considered the ideal genotypes due to their higher mean resistance reaction and a lower projection onto the axis, which signified their overall consistency in performance across the locations and years. These were followed by G7, a desirable genotype due to its low DS rating, lower projection onto the axis, and proximity to the ideal genotypes. Among the moderately resistant genotypes, G12, despite its 4.7 mean DS rating, projected the furthest on the axis, indicating low stability across the environments.

#### 3.3.2 Evaluation of factor E

The performance of the tested environments was evaluated using the GGE model-based discriminativeness *vs.* representativeness biplot (**Figure 3**). As previously mentioned, the single arrowhead line in the biplot indicated the average environment, AEC abscissa. During the first year (2017), Ludhiana exhibited the longest vectors and was therefore the most discriminating location regarding AB severity assessment amid other locations for that year. In the second year (2018), Jammu displayed the longest vectors followed by Palampur and Ludhiana. Among all locations, the vector for ICRISAT formed the smallest angle with the AEC during the first year, suggesting that it was the most representative of all test locations for that year. In the second year, Palampur demonstrated the least angle followed by Ludhiana and ICRISAT.

In addition, the repeatability of the tests in a particular location over the years was estimated by visualizing their relationship in the biplot. Environments that form acute angles are positively correlated, those forming right angles have no relation, and those forming obtuse angles have a negative correlation. The size of the angles determined the strength of the relationship. Here, each test location displayed a high positive correlation between the two years. Between the locations, Palampur showed a high correlation with Ludhiana, while the same was true between ICRISAT and Jammu.

From the perspective of identifying stable genotypes, the locations having high discriminative power, lower representativeness, and higher repeatability were favored over the others. With this in mind, Palampur and Ludhiana were the most preferable for detecting stable genotypes.

### 3.4 Identification of mega-environments

**Figure 4:**
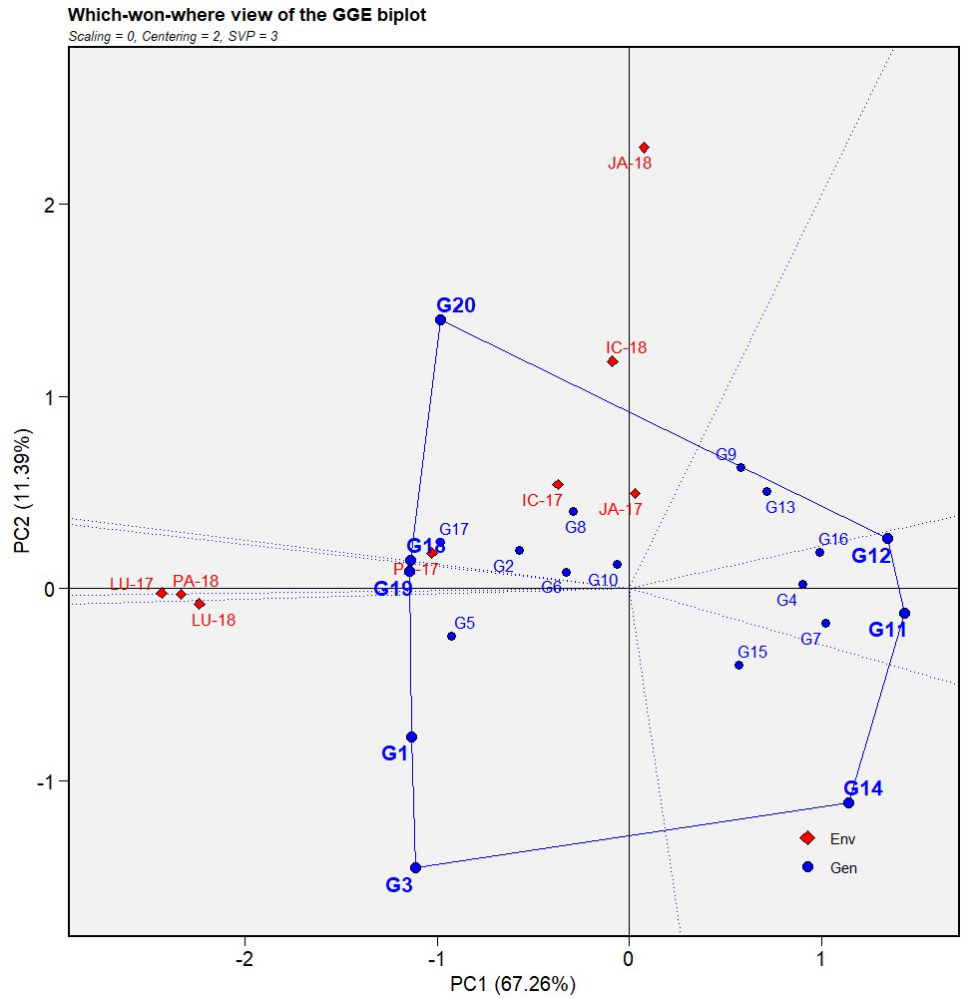
Which-won-where view of GGE biplot of IABN against AB severity across four locations during 2 years. The data were centered through environments (centering = 2) without scaling (scaling = 0). Locations are denoted as IC- (ICRISAT), JA- (Jammu), LU- (Ludhiana), and PA- (Palampur). Years are denoted as -17 (year 2017/2018) and -18 (year 2018/2019). IABN, International Ascochyta Blight Nursery; AB, Ascochyta blight.

The which-won-where view of the GGE biplot was utilized to identify environment-specific genotypes from the multi-environment testing (**Figure 4**). A polygon was constructed in the biplot, keeping either the best- or poorest-performing genotypes as the vertices. The sectorization of the winning genotypes was conducted by drawing a perpendicular line (equality lines) from the biplot origin to the sides of the polygon. The genotypes at the vertices were considered the most responsive, while those aggregating toward the origin were the least responsive genotypes for that location.

In our biplot, the genotypes occurring at the right side of the polygon (toward the convex hull) showed a more resistant reaction than those on the left side. Moreover, since these genotypes did not share the sector with any location, this implied that their performance was reasonably similar across all environments. Subsequently, the genotypes sharing sectors with particular locations were more suited to those environments than others.

For the first year (2017), the equality lines partitioned the four locations into two mega-environments, the first comprising three locations, ICRISAT, Jammu, and Palampur, while the second constituted Ludhiana alone. During the second year (2018), two mega-environments were again formed with the only difference being that both were constituted by two locations each, ICRISAT and Jammu in the first and Ludhiana and Palampur in the second.

## 4 Discussion

AB resistance screening almost always has a high margin for inconsistent disease expression across different environments (Rubiales et al., 2012). In India, the natural screening of chickpeas against AB on a large scale is difficult due to the lack of ample resources and the prevalence of highly variable agro-climatic conditions. Although chickpea is widely cultivated in central India, the historical trend of disease occurrence in these regions pointed toward a lack of ideal climatic conditions required for a consistent AB natural epidemic. Therefore, at the ICRISAT location, extensive screening of the experimental materials was undertaken under simulated conditions in controlled environment facilities. In our study, the initial screening of 160 chickpea genotypes reduced the set to 19 genotypes by rogueing out the highly susceptible entries. The final disease nursery (IABN) consisted of 20 genotypes including a highly susceptible check line ICC 4991 (G20). Several reports emphasize the importance of conducting preliminary screening followed by a selection of candidate genotypes prior to multi-location trials, allowing optimum resource allocation (Pande et al., 2013; Sharma et al., 2013; Parihar et al., 2017). The susceptible check not only is used in multi-environment testing to serve as a control against the tested genotypes but also plays an important role in indicating the disease pressure for any given location or period of time. Our check line constantly yielded a susceptible to highly-susceptible reaction toward AB in all environments tested, indicating a good disease pressure irrespective of the environments.

Thereafter, the multi-location testing conducted over 2 years revealed significant differences in the G, E, and G × E interaction, suggesting an overall diversity in the disease nursery (Pande et al., 2013). Majority of the tested genotypes produced variable reactions under different environments; a few even displayed completely contrasting responses, where the same genotype showed a highly resistant reaction in one location and a completely susceptible reaction in another location. Parihar et al. (2017), who made similar observations in yellow mosaic disease (YMD) of mung bean, suggested variability in the genotype or the causal organism or both together to be responsible for such differences. This is also supported by the fact that multi-environment trials of genotypes are inclined to have shifts in their relative ranking of G × E interaction (Alam et al., 2014).

The G × E interaction greatly impacts the selection process of genotypes suited for targeted environments (Pour-Aboughadareh et al., 2022). The GGE biplot analysis is a very useful method to evaluate the stability of the genotypes, environments, and formation of mega-environments (Yan and Kang, 2003) and thus the underlying G × E interactions. In our study, the genotype-focused mean *vs.* stability biplot (**Figure 2**) helped in identifying the genotypes with an overall moderate resistance reaction with stable performance across the tested environments. A total of three genotypes (two ideal and one desirable) were selected from the study for use in downstream resistance breeding programs. At the same time, the selection of suitable locations is also an important factor for maximizing genetic gains from selection based on G × E interactions (Yan et al., 2011).

During the multi-environment testing, prioritizing ideal over less-ideal locations conforms to optimizing resource allocation (Das et al., 2019). Our environment-focused discriminativeness *vs.* representativeness biplot (**Figure 3**) helped in delineating the suitability of the tested environments. Accordingly, Ludhiana and Palampur were determined to have higher stability than Jammu for detecting stable chickpea genotypes against AB. Here, the ideal location was explained by its higher discriminative power, lower representativeness, and good repeatability during the multi-environment testing. These findings were supported by Manjunatha et al. (2018), who reported the chickpea-growing regions of Punjab and Himachal Pradesh as hotspots for AB incidence.

The which-won-where biplot view of our GGE analysis led to the identification of different mega-environments formed by the test locations. The ability of the test locations to be grouped into distinct mega-environments suggested the presence of cross-over G × E interaction (Singh et al., 2020). In addition, they also serve the purpose of exploiting specific adaptations of genotypes within specific locations (Yan et al., 2011). In the present study, deviations in the mega-environment formation were observed between the years. This could be brought about by non-repeatable relationships between the tested location and the differences in AB severity as a result of genotypic and environmental variations (Krishnamurthy et al., 2017). The locations within each mega-environment displayed a high positive correlation and comparable performance by the genotypes within them. Also, the moderately resistant genotypes were not observed to fall into any of the sectors forming the mega-environment, which implied that the disease reactions for these genotypes were fairly similar in all the locations and years tested.

## 5 Conclusions

The present study showed the importance of undertaking multi-location trials, their subsequent G × E interactions, and stability analysis for the evaluation of genotypic resistance against AB disease. The GGE biplot method proved to be a useful method to delineate the underlying interactions between the genotype and environment. In addition to identifying moderately resistant genotypes having consistent performance across the tested environments, we could discriminate the test locations for their suitability for future screening trials. The controlled environment facility at the ICRISAT location was best suited for the preliminary screening of a large population of chickpea genotypes. Ludhiana and Palampur were identified as the ideal test locations for the natural screening of chickpeas against AB. These efforts establish the foundation for AB management in chickpeas through host–plant resistance.

## Data availability statement

The original contributions presented in the study are included in the article/supplementary material. Further inquiries can be directed to the corresponding author.

## Author contributions

MS conceived and designed the study. UR, SS, AB, and DB conducted the field trials and collected data. UC and MS were responsible for the analysis and interpretation of results. UC drafted the manuscript. MS provided critical inputs at various stages of the study and edited the manuscript. All authors contributed to the article and approved the submitted version.
